# Aquatic Eddy Correlation: Quantifying the Artificial Flux Caused by Stirring-Sensitive O_2_ Sensors

**DOI:** 10.1371/journal.pone.0116564

**Published:** 2015-01-30

**Authors:** Moritz Holtappels, Christian Noss, Kasper Hancke, Cecile Cathalot, Daniel F. McGinnis, Andreas Lorke, Ronnie N. Glud

**Affiliations:** 1 Max-Planck-Institute for Marine Microbiology, Biogeochemistry Group, Bremen, Germany; 2 MARUM—Center for Marine Environmental Science, University of Bremen, Bremen, Germany; 3 Institute for Environmental Sciences, University of Koblenz-Landau, Landau, Germany; 4 University of Southern Denmark, Institute of Biology and Nordic Center for Earth Evolution (NordCEE), Odense, Denmark; 5 Royal Netherland Institute for Sea Research (NIOZ), Yerseke, Netherlands; 6 IGB—Leibniz-Institute of Freshwater Ecology and Inland Fisheries, Experimental Limnology, Berlin, Germany; 7 Institute F.-A. Forel, Faculty of Science, University of Geneva, Geneva, Switzerland; 8 Aarhus University, Arctic Research Center, Aarhus, Denmark; 9 Scottish Association for Marine Science, Scottish Marine Institute, Oban, United Kingdom; 10 Greenland Climate Research Centre, Greenland Institute of Natural Resources, Nuuk, Greenland; Auckland University of Technology, NEW ZEALAND

## Abstract

In the last decade, the aquatic eddy correlation (EC) technique has proven to be a powerful approach for non-invasive measurements of oxygen fluxes across the sediment water interface. Fundamental to the EC approach is the correlation of turbulent velocity and oxygen concentration fluctuations measured with high frequencies in the same sampling volume. Oxygen concentrations are commonly measured with fast responding electrochemical microsensors. However, due to their own oxygen consumption, electrochemical microsensors are sensitive to changes of the diffusive boundary layer surrounding the probe and thus to changes in the ambient flow velocity. The so-called stirring sensitivity of microsensors constitutes an inherent correlation of flow velocity and oxygen sensing and thus an artificial flux which can confound the benthic flux determination. To assess the artificial flux we measured the correlation between the turbulent flow velocity and the signal of oxygen microsensors in a sealed annular flume without any oxygen sinks and sources. Experiments revealed significant correlations, even for sensors designed to have low stirring sensitivities of ~0.7%. The artificial fluxes depended on ambient flow conditions and, counter intuitively, increased at higher velocities because of the nonlinear contribution of turbulent velocity fluctuations. The measured artificial fluxes ranged from 2 - 70 mmol m^-2^ d^-1^ for weak and very strong turbulent flow, respectively. Further, the stirring sensitivity depended on the sensor orientation towards the flow. For a sensor orientation typically used in field studies, the artificial flux could be predicted using a simplified mathematical model. Optical microsensors (optodes) that should not exhibit a stirring sensitivity were tested in parallel and did not show any significant correlation between O_2_ signals and turbulent flow. In conclusion, EC data obtained with electrochemical sensors can be affected by artificial flux and we recommend using optical microsensors in future EC-studies.

## Introduction

In aquatic systems, the flux of oxygen across the sediment-water interface is an important proxy for quantifying the integrated benthic carbon mineralization and primary production of sediments [1]. Established approaches to quantify the benthic oxygen uptake in the field include oxygen microsensor profiles of surface sediments [2] and chamber incubations [3]. As an alternative approach, eddy correlation (EC) measurements were adapted from atmospheric sciences in 2003 [4]. The EC approach quantifies the oxygen flux in the turbulent boundary layer above the sediment by combining high frequency measurements of flow velocities and oxygen concentrations in the same sampling volume from which the instantaneous and the average oxygen flux can be calculated. EC measurements are non-invasive and allow flux measurements in various environmental settings where traditional methods fail. EC measurements were applied to estimate oxygen uptake of cohesive [5] and sandy [6] sediments, sea grass fields [7], hard bottom substrates [8], coral reefs [9], oyster reefs [10], and sea ice [11]. The possibility of long term EC measurements (hours to days) allows interpreting oxygen fluxes in the context of variable environmental conditions such as prevailing bottom water O_2_ [12], changing current velocity and light regime [6, 7, 10]. However, many of the measured oxygen fluxes show an extensive short term variability (minutes to hours) that is poorly explained by benthic community responses, but rather is related to transient adjustments of the O_2_ transport in BBL and sediment [13].

As EC measurements are increasingly applied in the field, there is still a need to identify the limits of the method and how best to apply the approach to get high-quality flux data. Recently, it was shown that non-steady state conditions with respect to hydrodynamics and mean oxygen concentrations in the benthic boundary layer can induce significant transient EC-fluxes that are not related to benthic exchange rates and that longer integration times averaging out such effects are required to obtain reliable results [13]. Also, the spatial heterogeneity of benthic communities should be considered when choosing the optimal measuring height above the seafloor [14]. Further, Donis et al. [15] described the effect of sensor misalignment and sensor response times on flux estimates, whereas Lorke et al. [16] discussed the effect of inappropriate coordinate rotation in data post-processing.

Here we report on the limits set by the use of electrochemical oxygen sensors for obtaining high quality EC flux measurements. While high frequency sampling of velocities is readily performed with a standard acoustic Doppler velocimeter (ADV) [17], the fast sampling of oxygen is more challenging. Electrochemical Clark type microsensors [18] have so far been the preferred tool for fast and precise sampling of turbulent O_2_ fluctuations—only very recently fast responding optical microsensors (optodes) have been suggested as a potential alternative as they are more robust and stable during long term deployments [19]. Electrochemical microsensors consume small amounts of oxygen as they measure the flux of oxygen diffusing from outside the sensor tip, through the membrane and to the sensing cathode where oxygen is ultimately reduced. The sensor signal is therefore a function of the sensor dimension, especially of the distance between the sensor tip and the cathode, which is determined by the membrane thickness [20]. This is taken into account as each sensor is individually calibrated. The distance for diffusive oxygen transport is enlarged by the diffusive boundary layer (DBL) at the outside of the sensor tip. The thickness of the DBL (δ_DBL_) depends on the size of the sensor tip but varies upon changes in flow velocities [18, 20, 21]. In stagnant water, the enlarged DBL increases the distance for diffusive oxygen transport causing reduced oxygen flux to the cathode and therefore a reduced signal, whereas in stirred water the signal is increased. The signal increase between stagnant and stirred conditions is known as stirring sensitivity and is typically in the range of 0.5–2% for Clark type microsensors [18, 22]. Ultimately, the stirring sensitivity is determined by the ratio of the variable distance (δ_DBL_) to the fixed distance (between tip and electrode) for the diffusive transport. There is no possibility to completely remove the stirring sensitivity since the sensor tip cannot be infinitely small and a fast sensor response requires a small distance between tip and electrode (i.e. the fixed diffusion distance). Although the stirring sensitivity constitutes an artificial correlation between ambient flow velocities and oxygen sensing, its effect on EC measurements has not been studied so far. The reason for this is that the signal change caused by the stirring sensitivity strongly decreases with increasing velocities [18, 20–22] and was therefore considered insignificant. In this study, we assess the effect of the stirring sensitivity on EC measurements theoretically and experimentally. In the first section, we present a theoretical analysis predicting significant EC fluxes caused by stirring sensitivity (from here on referred to as ‘artificial EC flux’) especially at increasing flow velocities. In a second part we experimentally quantify the artificial EC flux by measuring the correlation between the turbulent flow velocity and the signal of oxygen microsensors in a sealed annular flume where oxygen sinks and sources were eliminated (for all practical purposes). Finally we discuss the consequences for field measurements using a field study where both optode and electrochemical sensors were used, and discuss possible solutions for future EC measurements.

## Theory

In the turbulent boundary layer above the seabed, we define *u* as the longitudinal velocity along the main flow direction, whereas the transversal velocity *v* is perpendicular to *u* in the horizontal plane and the vertical velocity *w* is perpendicular to *u* and *v*. EC-fluxes are derived from high frequency measurements of concentrations and velocities recorded over time intervals much larger than the time scales of the largest eddies [23]. For flux calculations, the fluctuating concentrations and velocities are extracted by subtracting the mean values (calculated over long enough averaging times) from instantaneous values ($c'=c-\bar{c}$, $u'=u-\bar{u}$ and $w'=w-\bar{w}$). The vertical EC flux, which is usually of interest, is calculated from the correlation of the vertical velocity fluctuation *w’* and the concentration fluctuation *c’* [4]

$$EC\;flux=\bar{w'c'}$$ (1)

In the following, the concentration fluctuation *c’* caused by the stirring sensitivity of an electrochemical O_2_ sensor is modeled as a function of sensor specifications and flow conditions.

**Figure 1:**
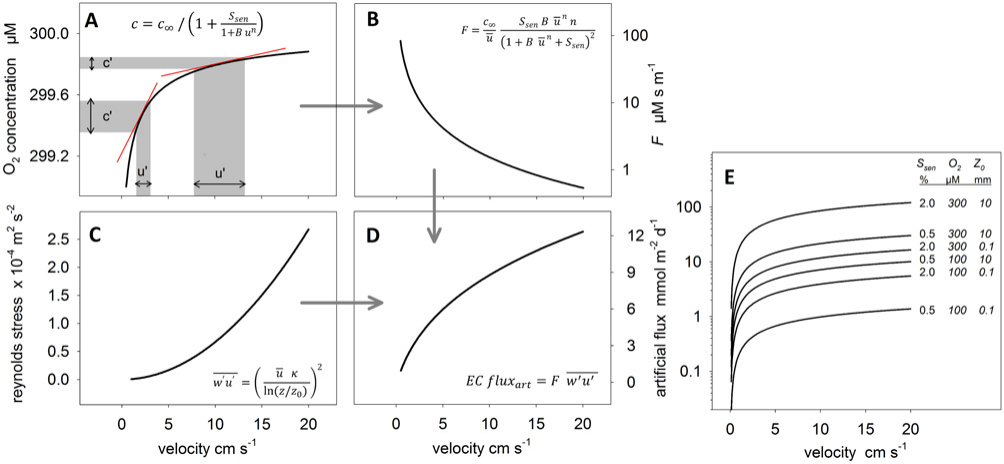
The predicted artificial flux as a function of stirring sensitivity and Reynolds stress. **A:** The O2 concentration measured by a microelectrode increases non-linearly with the current velocity, here shown for a sensor with a stirring sensitivity of 0.7%. As a first approximation, the fluctuating velocities (*u’*) around their mean ($\bar{u}$) can be transferred to the fluctuating concentrations (*c’*) around their mean ($\bar{c}$) using the slope of the function at $\bar{u}$ as a transfer factor (red lines in A). **B:** The first derivative of the transfer function, i.e. the transfer factor, decreases with velocity. **C:** The Reynolds stress in a logarithmic boundary layer increases as the square of the velocity, here assuming a roughness (*z0*) of 1 mm and a height above the seabed (*z*) of 15 cm. **D:** Applying equation 5, the Reynolds stress and the transfer factor can be combined to estimate the EC-flux from stirring sensitivity as a function of velocity. **E:** The artificial EC flux is plotted as a function of velocity according to equation 7 and as a function of *Ssen* (0.5–2%), *c∞* (100–300µM) and *z0* (0.1–10mm). The combination of parameter values is presented next to the functions.

The thickness of the DBL (δ_DBL_) at the tip of a microsensor depends on the flow velocity of the ambient water [18, 20–22]. High flow velocities reduce δ_DBL_ and thus the distance for the diffusive transport of O_2_ between the undisturbed water and the cathode, which causes an increase in the signal (electrical current) at the cathode and thus an apparent increase of measured O_2_ concentrations. Conversely, reduced flow velocities cause a decrease of the sensor signal which is highly non-linear as current velocities approach zero (Fig. 1A). This relationship is described for a membrane coated cathode sensor by the transfer function presented in Gust et al. [21]:

$$c=c_{\infty }/\left(1+\frac{S_{sen}}{1+B\;u^{n}}\right)$$ (2)

where the measured concentration *c* is a function of the longitudinal velocity *u* (in m s^-1^), the concentration at infinite velocities *c_∞_* (at δ_DBL_ = 0), and the stirring sensitivity of the sensor calculated as S_sen_ = c_∞_ / c_0_–1, where *c_0_* denotes the concentration measured in stagnant water. Values for the parameters *B* (79) and *n* (0.7) were derived by Gust et al [21] who fitted (2) to experimentally derived values of mean velocity and mean microsensor signal. Because Gust et al. used a different sensor type (membrane coated cathode sensor) with very high stirring sensitivities (10–55%), we repeated the fitting procedure with experimental data (see below) using a Clark-type O_2_ microelectrode with a stirring sensitivity of 0.7%. We found that the function fitted the flow response of the Clark type sensor very well using values of 30 and 0.65 for the parameters *B* and *n*, respectively. These values are used in the following and throughout the study. An example of the transfer function is shown in Fig. 1A for a Clark type O_2_ microelectrode with a stirring sensitivity of 0.7%, ambient O_2_ concentrations of 300 µM and for velocities ranging from 0.5–20 cm s^-1^. The deviation from the true O_2_ concentration strongly decreases with increasing velocities.

Gust et al [21] did not assess the behavior of the sensor signal with respect to the fluctuating component of the flow. However, the time for the readjustment of the diffusive boundary layer at the tip of the microsensor is in the order of milliseconds (δ_DBL_^2^/O_2_-diffusivity) suggesting a readjustment to changing velocities much faster than the response time of the sensor, which is in the range of 0.1–0.4s for fast responding Clark type O_2_ sensors applicable for EC measurements [22]. Consequently, turbulence induced changes of the sensor signal are instantaneous and fully captured within the limits set by the sensors response time. If the stirring sensitivity and the response time of a sensor are known, a time series of longitudinal velocities can be transferred into a time series of artificial concentration fluctuations using (2) and a function that accounts for the sensors response time (see method section below). Insertion of the derived artificial *c’* into (1) allows estimating the resulting artificial flux. This approach is denoted as MODEL 1 and is described in more detail in the method section.

To better understand the relation between flow conditions and the induced artificial flux we developed a generalized model, which estimates the artificial flux as a function of mean flow and turbulence statistics. For this, the response time of the electrochemical sensor is neglected, assuming it short enough to capture most of the turbulence associated O_2_ fluctuations, which is actually a prerequisite for EC measurements. By neglecting the response time, velocity fluctuations can be directly transferred into artificial concentration fluctuations using (2). To simplify matters, we now assume that for a velocity time series the fluctuating velocities (*u’*) around the mean velocity ($\bar{u}$) can be transferred into fluctuating concentrations (*c’*) using the slope of the transfer function at $\bar{u}$ as a transfer factor *F* (see red lines in Fig. 1A):

c'=Fu' (3)

A prerequisite for (3) is that $\bar{u}>u'$ and $\bar{c}>c'$. The transfer factor *F* for any mean velocity $\bar{u}$ is calculated from the first derivative of (2) with respect to velocity:

$$F=\frac{c_{\infty }}{\bar{u}}\frac{S_{sen}\;B\;{\bar{u}}^{n}n}{{\left(1+B\;{\bar{u}}^{n}+S_{sen}\right)}^{2}}$$ (4)

(see Fig. 1B). *F* is constant for constant *c_∞_* and over the averaging interval of *u*, which allows combining (1) and (3) to estimate the artificial EC flux

$$EC\;flux_{art}=F\;\bar{w'u'}$$ (5)

This relation is denoted as MODEL 2 in the method section below. It is evident from (5) that the flux caused by stirring sensitivity is directly proportional to the Reynolds stress ($\bar{w'u'}$), i.e. the correlation of vertical and longitudinal flow velocity fluctuations, which is readily measured with the ADV. From (5) it is also evident that the artificial flux is negative (i.e. downward directed) because the Reynolds stress in a turbulent boundary layer is usually negative [24] and *F* > 0. In other words, water parcels with an above average longitudinal velocity are predominantly downward directed (Reynolds stress) and induce at the same time an above average oxygen signal due to the stirring sensitivity. As a result, above average oxygen signals are associated with downward directed velocities, which is exactly what is expected for the true turbulent oxygen transport above oxygen consuming sediments. The predicted artificial flux from electrochemical O_2_ sensors, therefore, imitates benthic oxygen uptake.

Furthermore, the Reynolds stress and thus the artificial flux strongly depend on the mean flow velocity. The Reynolds stress in the logarithmic part of a turbulent boundary layer is equal to the square of the friction velocity u_*_ [24] which can be estimated from the law of the wall:

$$\bar{w'u'}=u_{*}{}^{2}={\left(\frac{u\;\kappa }{\ln(z/z_{0})}\right)}^{2}$$ (6)

where κ is the von Kármán constant (~0.41) and *z_0_* and *z* denote the effective bed roughness and the height above the seabed. At a fixed position above the seabed, the Reynolds stress increases with the square of the mean current velocity (Fig. 1C). In summary, the EC flux from stirring sensitivity can be approximated by inserting (6) and (4) into (5) which results in:

$$EC\;flux_{art}=\frac{c_{\infty }}{\bar{u}}\;\frac{S_{sen}\;B\;\;{\bar{u}}^{n}\;n}{{\left(1+\;B\;\;{\bar{u}}^{n}+S_{sen}\right)}^{2}}\;{\left(\frac{u\;\kappa }{\ln(z/z_{0})}\right)}^{2}$$ (7)

Assuming a sensor with a stirring sensitivity of 0.7% positioned 15 cm above the seabed, a bed roughness of 1 mm and ambient O_2_ concentrations of 300 µM results in an artificial EC flux increasing from 1 to 12 mmol m^-2^ d^-1^ as the current velocity increases from 0.5 to 20 cm s^-1^ (Fig. 1D). This counter intuitive response of the stirring sensitivity is caused by the quadratic increase of the Reynolds stress which overrides the decaying stirring effect at increasing velocities. According to (7) we also expect the artificial EC flux to increase proportional with ambient O_2_ concentrations (*c_∞_*) and with stirring sensitivity; the latter because *S_sen_* in the denominator of (7) is small compared to the other summands and can be neglected. Furthermore, the artificial flux increases with the square of the Reynolds stress (last bracket term) which does not only depend on the current velocity at the sensor position z but also on the roughness of the sediment (*z_0_*). Increased boundary roughness, therefore, increases the artificial flux as well. Fig. 1E shows the possible range of artificial EC fluxes that can be expected for a combination of realistic values for *S_sen_* (0.5–2%), *c_∞_* (100–300µM) and *z_0_* (0.1–10mm). Artificial EC fluxes may range from below 1 to more than 100 mmol m^-2^ d^-1^ illustrating the combined impact of environmental factors and stirring sensitivity.

The response of the predicted artificial flux to changing environmental conditions is similar to the expected responses of benthic oxygen uptake. It was shown that increased current velocities cause a thinning of the DBL at the sediment water interface [25, 26] as well as a flushing of permeable sediment [27], both transiently increasing the benthic oxygen uptake. Also, high oxygen levels in the bottom water increase the oxygen penetration depth and transiently increase the benthic oxygen uptake [28]. Because the artificial flux response is similar, it is extremely difficult to dissect the artificial flux from the true benthic uptake in field measurements. In the following experiment possible oxygen sinks and sources were eliminated to exclude oxygen gradients and ‘true’ fluxes in order to quantify the artificial flux induced by stirring sensitivity.

## Methods

### Flume

**Figure 2:**
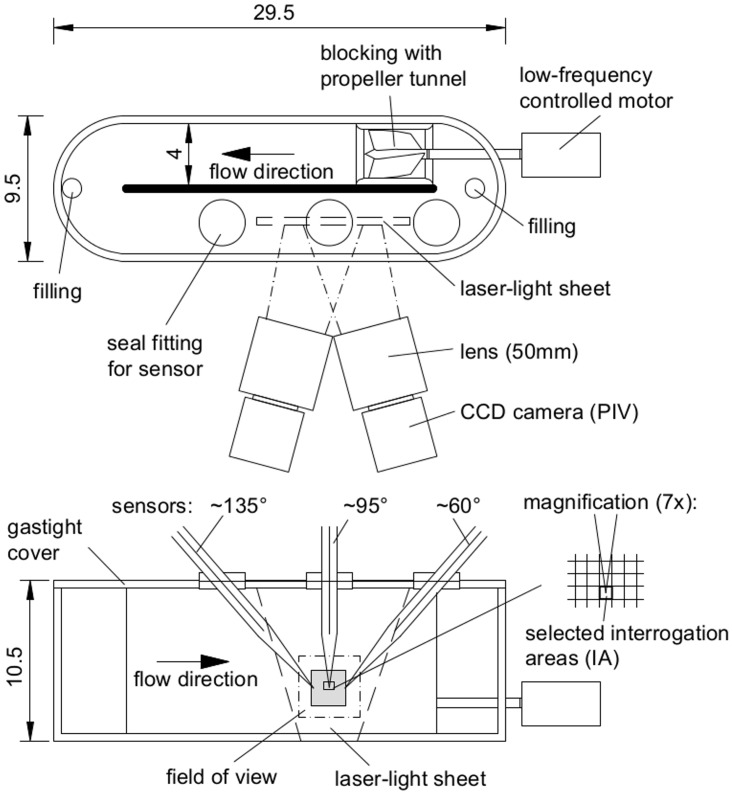
The experimental setup. Top view (top) and side view (bottom) of the flume. The electrochemical sensor was inserted either at ~135°, ~95° or ~60° inclination to the mean flow direction. Optode orientation was always at ~95°. All measures in cm.

Flume experiments were conducted in February 2013 at the Institute for Environmental Sciences, University of Koblenz-Landau Landau. Experiments were performed in a closed oval-shaped acrylic glass flume with cross-sectional width of 4 cm and height of 10 cm (Fig. 2). The total mean length of the flume was 54 cm consisting of two 20 cm long straight sections and two 180° curves with 0.25 cm inner and 4.25 cm outer radius. The test section was placed in the 2^nd^ half of a straight section. The fluid flow was induced by a propeller (Ø 3 cm) situated in a blocking wall at the start of the opposing straight section. The propeller was driven by a motor and mean flow velocities of up to 20 cm s^-1^ were generated by applying voltages between 0 V and 4 V DC. Occasionally, wavelike flow was induced by superposition with 0.3 V AC at a frequency of 0.3 Hz. The flume was completely sealed with an acrylic glass cover. Sensors were inserted through 40 mm long glass tubes situated in rubber seal fittings. The fittings were placed at different positions along the test section and allowed positioning the sensors with inclinations to the main flow direction of ~60°, ~95° and ~135°.

### Velocity measurement

Using stereoscopic particle image velocimetry (PIV) [29] instantaneous three-dimensional flow velocities were measured in a 2.5 mm thin, 4 cm long, and 4 cm high laser-light sheet, which was aligned with the longitudinal and vertical axes of the straight flume section. To avoid the sensor reflecting the laser light, the tip of the sensor was placed approximately 2 mm in front or behind the laser light sheet. Neutrally buoyant seeding particles (20 µm polyethylen, Dantec Dynamics, Denmark) were added to the flume water. Particles were illuminated by a double pulsed 532 nm laser at a frequency of 7.4 Hz and recorded by two 4 megapixel CCD cameras. The pulse length was 4 ns and the time between pulses was adjusted between 3 ms and 9 ms for an optimal observation of the particle displacement in dependence of the flow velocity. A two frame adaptive correlation analyses was conducted for both cameras to estimate two-dimensional velocity vectors for 32 x 32 pixel interrogation areas with 50% overlap. A field of 3-dimensional velocity vectors with a spatial resolution of 1.15 mm (longitudinal) and 1.17 mm (vertical) was received by intersecting the two 2-dimensional vectors. Velocity time series were extracted from the instantaneous velocity vectors at the interrogation area closest to the tip of the respective sensor.

### Oxygen measurement—Electrochemical sensor

**Figure 3:**
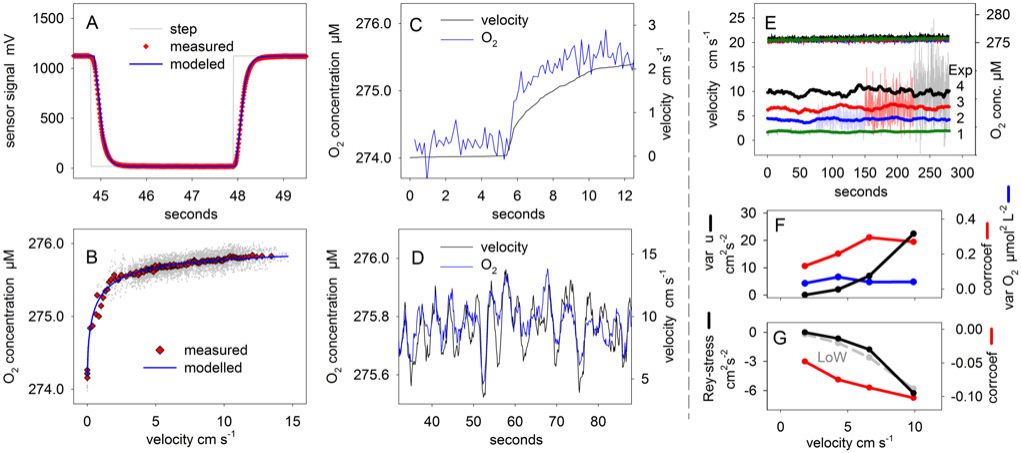
Characteristics of the electrochemical sensor and flow statistics. **A:** The sensor response time was measured upon a step change in O_2_ saturation from 100 to 0%. Equation 9 was used to model the sensors response upon a step change. **B:** Several compiled and equation 2 was fitted to the compiled data set to determine the stirring sensitivity. Grey dots are individual data points, red dots are 2–20 bin averages. **C+D:** The response of the electrochemical sensor to changing flow velocities was measured after an abrupt increase of the velocity from 0 to 2.2 cm s^-1^ and for an oscillating velocity of 0.3 Hz. **E** depicts the mean velocities (20 s running average) of Exps. 1–4 underlaid with snap shots of instantaneous velocities. In the upper part the O_2_ concentrations of the electrochemical sensor show that the drift of the sensor is negligible over the course of the 5 min measurements. **F** shows the variance of the O_2_ concentration (blue line), the variance of the longitudinal velocity (black line) and the correlation coefficient of the *u’* and *c’* time series (red line) as a function of flow velocity for Exps 1–4. **G** shows the correlation coefficient of the *w’* and *c’* time series (red line) and the measured Reynolds stress (black line). The latter is compared to estimates from the Law of the Wall (LoW, grey dashed line) for the flow at 15 cm above the seafloor and assuming a roughness length scale of 2.8 cm.

Clark type electrochemical O_2_ sensors with an internal reference and guard cathode [18] are widely applied for EC measurements and were therefore used in this study. This sensor type can be optimized for different measuring characteristics (stirring sensitivity, response time) depending on the tip size, membrane thickness and distance from tip to electrode [18]. In this study, all measurements were done with the same electrochemical microsensor which was custom built at the University of Southern Denmark and had a tip diameter of ~8 µm and an opening of 1–2 µm. The distance from tip to cathode was approx. 20 µm with a 10 µm thick silicone membrane in between. A response time (*τ_90_*) of 0.4 s was determined from the response of the sensor to a step change in concentration (Fig. 3A). The sensor was mounted in a sensor holder and connected to a pico amperemeter designed for EC-measurements (Unisense). The signal was passed to an analog/digital converter (DaqPad, National Instruments) and recorded with a sampling frequency of 1 kHz on a Laptop using the software LabView (National Instrument). Amperemeter, converter and Laptop were powered by 12V batteries and the metal casings of sensor holder and pico amperemeter were electrically grounded to minimize the 50 Hz noise from the 220 V electricity grid.

### Stirring sensitivity

The stirring response of the electrochemical sensor was determined in the flume during the increase of the mean flow velocity from 0 to 2.2 cm s^-1^ which caused the sensor signal to increase by ~0.5% (Fig. 3C). In addition, the stirring response of the sensor was recorded during low frequency (0.3 Hz) sinusoidal changes in current velocities between 1 and 9 cm s^-1^ (mean velocity 5.1 cm s^-1^) and between 3.4 and 14 cm s^-1^ (mean velocity 9.3 cm s^-1^) (Fig. 3D). The sensor was positioned at ~60° inclination to the mean flow direction (Fig. 2). The data sets were combined to describe the change of O_2_ concentrations over a large range of velocities (Fig. 3B). The best fit of the transfer function (2) to the compiled data set resulted in values (±SE) of 30 (±4.9) and 0.65 (±0.03) for the parameters *B* and *n*, respectively and in a stirring sensitivity of *S_sen_* = 0.7% (±0.01%).

### Oxygen measurement—optodes

Oxygen optodes were fast-responding needle-type O_2_ optodes (PyroScience GmbH, Germany) with a spherical tip diameter of 50 µm and a measured response time of 0.2 s. The optodes used in this study were optimized for fast response and their signal is based on dynamic fluorescence quenching, where no rate limiting binding occurs and only the diffusion of oxygen is limiting the response time of the optode. To ensure a fast response time the optodes had no optical isolation. Optodes should not exhibit a stirring sensitivity as they do not consume O_2_. Sensor control was performed by a PyroScience OEM module, where settings and calibration were done while connected to a Laptop PC (Firesting II software, PyroScience). To reduce noise during measurements, the OEM module was operated in stand-alone mode, disconnected from the PC and powered by an independent battery source. O_2_ was measured at a frequency of 5 Hz. The signal was recorded using the analog output of the OEM module which gives a continuous output signal (0–5V) changing with the measuring frequency. The analog output was recorded as described above for the electrochemical sensor using a second channel of the same analog/digital converter.

### Sensor calibration

The optode sensor was calibrated by a two-point calibration performed in anoxic (dithionite solution) and 100% air saturated distilled water, respectively. Temperature dependent O_2_ saturation concentrations were calculated according to [30]. The electrochemical sensor was calibrated in a similar way with the exception that the upper calibration point was determined when the sensor was situated in the flume and by using the concentrations determined by the optode.

### Experimental setup

Prior to the experiments, all components of the flume were carefully rinsed with ethanol and washed with ultrapure water (MilliQ). Ultrapure water was bubbled with air at room temperature (21.0°C) until fully saturated and carefully filled into the flume removing the entire gas phase from the flume. Any remaining bubbles were removed with a syringe and a long bend needle that was inserted through one of the filling ports. Seeding particles were added and the filling ports were sealed with rubber stoppers. The sensors were inserted through the glass tubes and the water volume replaced by the sensors was pressed through the gap between sensor casing and glass tube avoiding excess pressure in the flume. The flow velocity was adjusted to ~5 cm s^-1^ for at least 30 min to allow thorough mixing and to ensure the absence of any gas bubbles. For each experiment, the flow was first adjusted to the targeted velocity and kept running for approximately 10 min. Then, flow velocity and oxygen concentrations were measured simultaneously for 5 min. Synchronized data sampling was ensured by recording the trigger signal of the PIV laser simultaneously with the signal of the electrochemical and optode sensors, using a sampling frequency of 1 kHz. For a total of 18 experiments the flow velocity was adjusted between 1.7 and 19.2 cm s^-1^, and 3 different orientations of the electrochemical sensor were tested with inclination angles of ~60°, ~95° and ~135° with respect to the main flow direction (Fig. 2). In 3 experiments wavelike flow was induced, whereas in all other experiments the motor was driven by constant voltages. An overview of all experiments is presented in Table 1. In 7 experiments, O_2_ was additionally measured by optodes. Although performed simultaneously with the electrochemical sensor, optode measurements are listed as separate experiments, because the velocity time series was extracted at the optode tip, located at a different position in the flume. When recording optode signal and velocity simultaneously, significant spikes in the optode reading appeared due to optical interference whenever the laser pulse (2 x 4 ns at 7.4 Hz) overlapped with the optode measurements (20 ms at 5 Hz) which was found for approximately 10% of the optode data. These data were discarded. The electrochemical sensor was not affected by the laser. For both sensors we did not observe any interference with seeding particles and no flow induced vibrations of the sensors were visible in the PIV recordings.

**Table 1:** Summary of experimental settings and measured fluxes.

**Exp. no**	**wave Hz**	$\bar{u}$ cm s^−1^	$\bar{w'u'}$ × 10^−4^	$\bar{u'u'}$ m^2^ s^−2^	**sensor**		**flux longitudinal**		**flux vertical**	
					type	angle	mmol m^−2^ d^−1^	p-value	mmol m^−2^ d^−1^	p-value
1	no	1.8	−0.001	0.05	el-chem.	57°	4.5	10^−09^	−1.8	0.03
2	no	4.3	−0.643	2.06	el-chem.	60°	65.9	10^−20^	−21.5	10^−04^
3	no	6.6	−1.772	7.02	el-chem.	61°	135.9	10^−43^	−31.7	10^−05^
4	no	9.9	−6.270	22.45	el-chem.	61°	223.9	10^−36^	−69.5	10^−06^
5	no	2.7	−0.042	0.39	el-chem.	92°	23.2	10^−21^	3.3	* 0.16
6	no	4.3	−0.177	1.30	el-chem.	95°	30.2	10^−14^	19.4	10^−07^
7	no	5.8	−0.706	2.95	el-chem.	95°	56.1	10^−23^	5.6	* 0.29
8	no	8.5	−2.272	7.51	el-chem.	95°	83.8	10^−18^	−0.4	* 0.96
9	no	11.9	−4.418	15.52	el-chem.	97°	107.4	10^−07^	30.3	* 0.11
10	no	14.6	−8.137	29.53	el-chem.	97°	191.6	10^−15^	−52.3	0.01
11	no	19.2	−16.303	57.02	el-chem.	96°	232.3	10^−12^	−31.6	* 0.32
12	no	2.4	0.009	0.35	el-chem.	133°	10.2	10^−06^	5.8	10^−03^
13	no	4.6	−0.391	3.69	el-chem.	137°	37.9	10^−05^	7.9	* 0.33
14	no	6.2	−1.258	8.80	el-chem.	137°	40.4	10^−04^	48.6	10^−07^
15	no	7.4	−2.593	14.86	el-chem.	139°	67.4	10^−06^	25.0	0.04
16	0.3	2.8	−0.196	0.78	el-chem.	95°	31.3	10^−30^	8.7	10^−04^
17	0.3	4.5	−0.131	3.76	el-chem.	97°	89.7	10^−53^	35.5	10^−16^
18	0.3	7.3	−1.997	10.60	el-chem.	98°	176.3	10^−57^	19.6	0.04
1op	no	1.7	0.001	0.05	optode	94°	−0.1	* 0.93	−1.6	* 0.05
3op	no	6.2	−1.964	7.45	optode	98°	5.9	* 0.45	4.1	* 0.55
4op	no	9.6	−5.524	23.72	optode	100°	8.0	* 0.56	7.0	* 0.58
12op	no	2.4	0.014	0.29	optode	94°	5.6	* 0.05	1.3	* 0.60
13op	no	5.2	−0.581	2.82	optode	97°	2.2	* 0.76	−5.1	* 0.42
14op	no	7.1	−1.802	6.76	optode	98°	−10.2	* 0.20	17.7	* 0.13
15op	no	8.6	−3.040	12.04	optode	98°	15.5	* 0.43	−1.4	* 0.93

Experiments were performed at constant voltages and occasionally with an undulating voltage at 0.3 Hz. Mean velocity $\bar{u}$, Reynolds stress $\bar{w'u'}$, and variance $\bar{u'u'}$ measured at the tip of the sensors are listed. The orientation of the electrochemical sensors varied and is expressed as the angle between mean flow direction and sensor. The longitudinal and vertical fluxes are given with the corresponding probability of the null hypothesis (i.e. zero correlation between velocity and O_2_ time series). Extremely low probabilities are presented in scientific notation. The asterisk marks non-significant fluxes (p>0.05).

### Data processing

The optode data were despiked by first identifying the overlap of optode reading and trigger signal, and then removing the peak and interpolating between neighboring values. O_2_ signals were recorded with 1 kHz and were first filtered using a running average with a window size of 100 samples to reduce any 50 Hz noise from the 220 V electricity grid. Subsequently O_2_ data were extracted according to the trigger signal of the PIV (7.4 Hz), which allowed correlating the oxygen reading of the electrochemical sensor and the optode with the velocities at the respective sensor tips.

Oxygen and velocity data were processed much the same way eddy correlation data are usually processed [23], with the exception that fluxes were calculated for both, vertical and longitudinal directions. For the latter, (1) was modified to $EC\;flux=\bar{u'c'}$. To prevent the projection of the longitudinal flux into the vertical direction any misalignment between the x-direction of the PIV system and the mean flow direction were minimized by applying a double coordinate rotation. Rotation of the velocity field was performed around the z-axis and around the y-axis, until mean transversal velocity ($\bar{v}$) and mean vertical velocity ($\bar{w}$) were zero, and mean longitudinal velocity ($\bar{u}$) was maximized. A running average with a window of 20 s was subtracted from instantaneous longitudinal (*u*) and vertical velocities (*w*) and from concentrations (*c*) to calculate fluctuating velocities (*u’, w’*) and concentrations (*c’*). To account for the response time of the electrochemical sensor, the correlations $\bar{w'c'}$ and $\bar{u'c'}$ were calculated as a function of time lag (up to 1.3 s) between the time series [23, 31], and the strongest correlation was used to calculate the flux, which was usually found at time lags below 0.4 s. The significance of the correlation (i.e. of the flux) was determined by first normalizing the correlation with the product of thestandard deviations of *u* and *c*:

$$corrcoef=\bar{u'c'}/\sqrt{\bar{u'u'}\;\bar{c'c'}}$$ (8)

here shown for the longitudinal direction. The resulting correlation coefficient was subsequently evaluated by calculating the probability of receiving the same correlation coefficient from random data sets (corrcoef function in Matlab) [13]. The threshold for a significant flux was set to 5%. Besides the mean flux, the cumulative flux, the cross-correlation function and the cumulative cospectra were also calculated.

### Modeling

Artificial EC-fluxes were modeled in two different ways. MODEL 1: From measurements the mean O_2_ concentration and the instantaneous longitudinal velocities were inserted in (2) to derive a time series of instantaneous concentrations. For MODEL 1, we considered the response time of the sensor (0.4 s) which attenuates the stirring response at high frequency velocity fluctuations. For this, the time series of instantaneous concentrations (č_1_, č_2_,.., č_n_) was low pass filtered and transferred into a time series of attenuated concentration fluctuations (c_1_, c_2_,.., c_n_) using a recursive approximation:

$$c_{n}=0.4\;c_{n}{}_{-1}+0.6\;(0.6\;{č}_{n}+0.4\;{č}_{n-1})$$ (9)

Equation 9 was applied to the step change used to determine the response time of the sensor (Fig. 3A). The factors (0.4 and 0.6) were derived from best fitting the modeled sensor signal to the measured signal. Finally, to calculate the artificial EC-flux, the time series of modeled concentrations were treated just as the measured concentrations described above. MODEL 2: Equation 5 was applied. For this the Reynolds stress was calculated from the velocity time series, and the factor *F* was calculated from mean longitudinal velocities, mean O_2_ concentrations and the determined stirring sensitivity of the sensor (0.7%, see above). For predicting longitudinal fluxes (5) was modified to: $EC\;flux_{art}=F\;\bar{u'u'}$ and the variance of the longitudinal velocity $\bar{u'u'}$ was inserted.

## Results

### Turbulence statistics and correlations

In the following, results of Experiments 1–4 (Table 1) are presented in detail. The electrochemical sensor was tilted ~60° with respect to the mean flow velocity (Fig. 2) representing the typical orientation often used in field measurements [4, 5]. The sensor signal was recorded at 4 different mean velocities of 1.8, 4.3, 6.6, and 9.9 cm s^-1^ (Fig. 3E). The variance of the instantaneous longitudinal velocity increased significantly with mean velocity from 0.05 to 22.5 cm^2^ s^-2^ (Fig. 3F), while the Reynolds stress changed from -0.001 to -6.2 cm^2^ s^-2^ (Fig. 3G). The variance of the O_2_ signal did not respond to the mean velocity but was constant at 0.05 µmol^2^ L^-2^, suggesting a considerable amount of noise in the recording—most likely electrical noise—which masked the stirring induced signal. However, the correlation coefficient, as calculated from (8), increased with longitudinal velocity from 0.13 to 0.29 (Fig. 3F), meaning that 13–29% of the variance of the O_2_ fluctuations can be explained by the longitudinal velocity fluctuations. This is significant, considering the contribution of the electrical noise to the O_2_ variance. High O_2_ concentrations also correlated with downward directed velocity as shown by the negative correlation coefficient for the vertical direction (Fig. 3G), which increased from -0.05 to -0.1. All correlations were highly significant (p-value in Table 1).

### Artificial flux as a function of velocity

**Figure 4:**
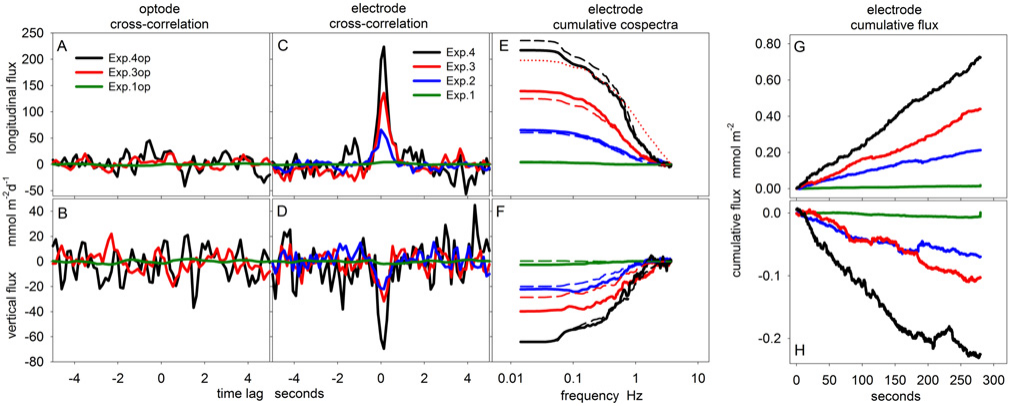
Cross-correlation, cumulative co-spectra and cumulative fluxes. **A-D** show the cross-correlation analysis of O_2_ measurements from optode and electrochemical sensor with the longitudinal and vertical velocity, respectively. **E+F** depict the cumulative cospectra of O_2_ concentrations (electrochemical sensor) and longitudinal and vertical velocity, respectively. The dashed lines represent simulated cospectra (MODEL 1). The dotted red line (**E**) shows the simulated cospectra for Exp. 3 without applying equation 9. **G+H** show the measured cumulative fluxes in the longitudinal and vertical direction. Please note: fluxes from Exp. 1 are significant but too low to be visualized here.

The artificial flux recorded in Experiments 1–4 is further analyzed using statistical measures commonly applied in EC-flux measurements [4, 23]. Fig. 4 shows the cross-correlation function of the velocity and O_2_ time series over a time lag of ± 5 s. O_2_ concentrations measured by the optode did not result in distinct peaks in the cross-correlation analysis (Fig. 4A+B) and, accordingly, the fluxes were not significant. In general, all optode measurements resulted in non-significant correlations in the vertical as well as in the longitudinal direction (see p-values in Table 1). For the electrochemical sensor the cross-correlation analysis reveals distinct peaks near the zero time lag (Fig. 4C+D) representing significant fluxes in the longitudinal direction of 4 to 220 mmol m^-2^ d^-1^ and in the vertical direction of −2 to −70 mmol m^-2^ d^-1^ (Table 1). The correlation rapidly decreases at larger time lags and its scatter can be interpreted conservatively as the range of possible correlations from randomized time series. This scatter of non-significant correlation was comparable between electrochemical and optode derived O2 concentrations.

All cumulative fluxes exhibited linear slopes (Fig. 4G+H) and the cumulative co-spectra were comparable to those from MODEL 1 (Fig. 4E+F). The cumulative co-spectra indicate that frequencies between 1 and 0.1 Hz contributed most to the flux (Fig. 4E+F). At high frequencies, the flux was attenuated by the response time of the sensor (0.4 s), which was well reproduced by MODEL 1. An example of MODEL 1 without considering the response time (i.e. without equation 9) shows significantly higher contributions at high frequencies (red dotted line in Fig. 4E). At low frequencies, a plateau was reached usually before the cutoff frequency, determined by the 20 s averaging window (see data processing above). Increasing the size of the averaging window did not cause a change of the flux.

### Artificial flux as a function of sensor orientation

**Figure 5:**
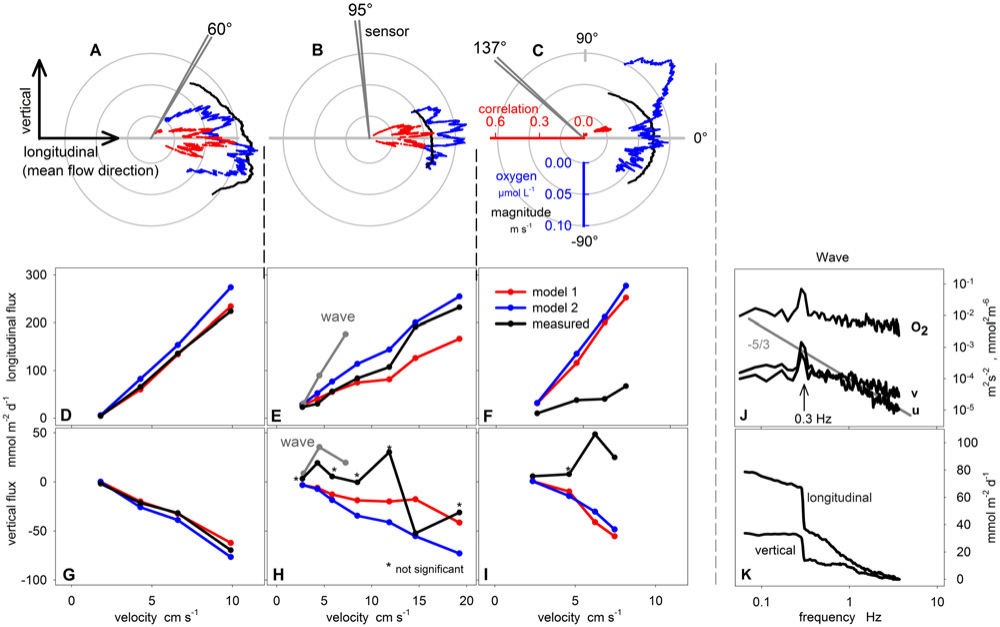
The electrochemical sensor: The artificial flux as a function of sensor orientation. **A-C:** The polar plots show flow magnitude (black) and oxygen anomaly (blue) as well as the correlation coefficient (red) as a function of flow direction and for different sensor orientations of 60° (Exp. 4, **A**), 95° (Exp.7, **B**), 135° (Exp. 14, **C**). Oxygen and magnitude share the same axis. **D-I:** For each sensor orientation, fluxes in longitudinal and vertical direction are summarized underneath the respective polar plot as a function of velocity. Measurements (black) are compared with MODEL 1 (red) and MODEL 2 (blue). Experiments with wavelike flow are marked in grey. **J** shows the power spectra of longitudinal and vertical velocities, and O_2_ signal under wavelike flow conditions (Exp. 17) with peaks at a frequency of 0.3 Hz. The grey line depicts the -5/3 slope of the inertial subrange. **K** shows the corrsponding cumulative co-spectra of the longitudinal and vertical fluxes.

Fig. 5 summarizes measured and modeled fluxes as a function of sensor orientation, flow velocity, and flux direction (longitudinal and vertical). To illustrate the effect of the sensor orientation, results from Exps. 4, 7, and 14 were analyzed in detail using polar coordinates (Fig. 5A-C). The processing was as follows: after converting the velocity to polar coordinates (i.e. angle and magnitude), the magnitude of the velocity and the corresponding O_2_ fluctuation were sorted by angle. Thereafter, mean velocity, mean O_2_, and flux were calculated for a window of 200 samples, which was shifted stepwise from low to high angles. Fluxes were weighted by the number of evaluated fluxes per degree, so that their average was equal to the flux calculated for the full data set. For better comparison, fluxes were divided by the product of the standard deviations of O_2_ and magnitude analog to (8). An offset was added to the running mean of the O_2_ fluctuations to avoid negative values, which cannot be presented in polar plots.

Sensor orientation was affecting both, the O_2_ signal and the correlation coefficient. When the sensor was tilted ~60° (Fig. 5A), highest O_2_ readings were found at flow directions of −10° to −30°, which is nearly perpendicular to the sensor orientation. O_2_ and velocity were significantly correlated for directions ranging from -26° to 20° (only correlations with p<0.05 are shown) with the number of high correlations in the downward direction exceeding those in the upward direction. The same data set analyzed in Cartesian coordinates shows a downward directed vertical flux (Fig. 5G, black line). At this sensor orientation, the vertical flux was always significant and downward directed (i.e. negative) and, similar to the longitudinal flux, shows an almost linear increase with flow velocity that agrees well with the simulated fluxes from MODEL 1 and MODEL 2 (Fig. 5D+G).

When the sensor was tilted ~95° (Fig. 5B), high O_2_ readings were found at flow directions ranging from -5° to 10° which is again perpendicular to the sensor orientation. O_2_ and velocity were significantly correlated for directions ranging from -21° to 32° with an overall larger proportion in the upward direction. The same data set analyzed in Cartesian coordinates shows a vertical flux that was upward directed and non-significant (Table 1, Exp. 7). In general, vertical fluxes at this sensor orientation show erratic behavior, being up- or downward directed and mostly non-significant (Fig. 5H, Table 1). This erratic behavior was not captured by the models. The longitudinal fluxes, however, were still significant and fairly predictable by the models (Fig. 5E).

When the sensor was tilted 135° (Fig. 5C), high O_2_ readings were found at flow directions ranging from 12° to 60° with a pronounced peak at 40° which is again perpendicular to the sensor orientation. O_2_ and velocity were significantly correlated only in a narrow range between 17° and 27° and the correlation coefficient was reduced compared to the previous orientations. The same data set analyzed in Cartesian coordinates shows a vertical flux that was significant and upward directed (Table 1, Exp. 14). At this sensor orientation, all vertical fluxes were upward directed and mostly significant (Fig. 5I, Table 1). Longitudinal fluxes were significant as well (Fig. 5F). However, in contrast to previous orientations they were lower than predicted by the models. The reason is that the correlation was reduced and only significant in a narrow sector between 17° and 27°, representing only a small fraction of the measured time series.

### Artificial flux at wavelike velocity

In experiment 16, 17, and 18 a wavelike velocity was generated. In the velocity spectrum, a distinct peak at 0.3 Hz marks the increased energy input of the wavelike motion, which is reflected also by a peak in the O_2_ spectrum (Fig. 5J). In combination this causes a sudden increase in the cumulative co-spectra at 0.3 Hz (Fig. 5K) suggesting an over proportional impact of the wavelike velocity on the O_2_ signal of the electrochemical sensor. Indeed, the resulting fluxes in longitudinal and vertical directions were strongly increased compared to fluxes measured at similar mean velocities (Fig. 5 E+H).

## Discussion

### Artificial fluxes from stirring sensitivity

Both, theoretical considerations and experiments clearly indicate that the stirring sensitivity of electrochemical O_2_ sensors can significantly bias the O_2_ flux estimated from eddy correlation measurements. Experimental results supported the theory which predicts increasing artificial fluxes for increasing Reynolds stress, i.e. for increasing flow velocities and/or bed roughness. This is the main outcome of the study and best shown by the correlation (i.e. the artificial flux) in the longitudinal direction (Fig. 5D-F) which is always significant and which always increases with current speed irrespective of the sensor orientation. This sensor behavior was well explained with a simple mechanistic model based on only 3 parameters (*S_sen_*, *B* and *n*) which enables more general testing of sensors with different configuration. The fraction of the artificial flux that was projected into the vertical direction was sensitive to the sensor orientation. For the typical sensor orientation (60°), the artificial vertical flux was well predicted by both MODEL 1 and MODEL 2 suggesting that the large range of predicted fluxes from below 1 to more than 100 mmol m^-2^ d^-1^ (Fig. 1E) can occur.

Because stirring sensitivity was not considered in EC measurements so far, the sensor parameter *S_sen_* was hardly mentioned in method descriptions, and no standardized quantification was developed. Typically, the stirring sensitivity is measured along with the 100% calibration of the sensor by switching off and on the stream of air bubbles in the calibration chamber, which leads to stagnant and undefined turbulent flow conditions, respectively. The electrochemical sensor used in this study exhibited a stirring sensitivity of not more than 0.7% which results in rather conservative estimates of the measured artificial flux. Furthermore, the long response time of the sensor of 0.4 s led to a significant attenuation of the signal (Fig. 4E). In practice, sensors are often selected for fast response times, because fast sensors capture a larger frequency range of the flux and produce a less attenuated signal. However, due to their smaller membrane-cathode distance, fast sensors usually exhibit higher stirring sensitivities. The optimal sensor design is therefore a tradeoff between fast response time and low stirring sensitivity.

In the flume experiments, artificial fluxes were high even for low velocities of a few cm s^-1^. The reason for this was the high turbulence intensity at the sensor tip because of its close proximity to the flume wall (2 cm). To relate the turbulent conditions in the experiment to those found in the field we matched the Reynolds stress measured in the experiments to those derived from the law of the wall (6) assuming the same current velocities at the sensor tip, but a sensor position 15 cm above the seafloor (Fig. 3G). The best fit was found for a bed roughness (*z_0_*) of 2.8 cm, which is in the range of roughnesses found for e.g. sediment ripples [32], gravel beds [33], oyster reefs [10] or coral reefs [34]. Besides the mean flow velocity, it is therefore important to consider the bed roughness, which also determines the Reynolds stress and thus the magnitude of the artificial flux.

The polar plots revealed highest O_2_ values at flow directions perpendicular to the sensor direction (Fig. 5A-C) suggesting that the sensor tip experiences maximum shear stress and minimum DBL thickness at this flow angle. This compares well with the well-studied case of fluid flow around a solid sphere where the shear stress peaks at surfaces that are oriented perpendicular to the flow [35], but rapidly decreases as soon as areas are shaded from the flow. For the typical sensor orientation (60°), the artificial flux was well predicted by the models because the sensor tip was never shaded from the flow (Fig. 5A) and the deformation of the DBL was a function of velocity only. At other sensor orientations the tip was increasingly shaded, first from downward directed flow at 95° orientation (Fig. 5B), which caused erratic vertical fluxes, and then from most of the flow at 135° orientation (Fig. 5C), which caused also low longitudinal fluxes. The different behavior of the artificial vertical flux with respect to flow direction could be used in field measurements to indicate if the measured flux is affected by stirring sensitivity (see below). In general, changes in flow velocity and direction cause a variable and sometimes erratic behavior of the artificial flux which could explain some of the short term variability of EC fluxes that is often observed in field studies (e.g. [8, 12]).

### Implications for field studies

Our results show that the artificial flux increases with turbulent intensity, i.e. with increasing current velocity and/or bed roughness (Fig. 1E) which is found e.g. in tidally influenced shallow waters or fast flowing rivers. The possible impact of such elevated artificial fluxes on EC measurements is enlarged by the decreasing potential to resolve the true benthic O_2_ fluxes in such turbulent environments. This is because, high turbulent mixing leads to the erosion of mean vertical O_2_ gradients in the boundary layer [13, 36, 37] and thus to smaller amplitudes of true O_2_ fluctuations, of which an increasing part may fall below the resolution of the O_2_ sensor. In addition, the fraction of high frequency eddies contributing to the turbulent O_2_ transport increases (i.e. the co-spectrum shifts towards high frequencies) and thus a larger part of the O_2_ flux is attenuated by the low pass behavior of the O_2_ sensor. In summary, an increased artificial flux in combination with a decreased potential to resolve the true O_2_ flux suggests a growing error for EC measurements in highly turbulent environments. If wave motion is added to this scenario the error is likely to increase even more (Fig. 5E+H).

The described artificial fluxes behave like several observed responses of the EC flux to changing environmental conditions in the field. Artificial fluxes increase with velocity, O_2_ concentration and boundary roughness (see equation. 7)—responses that are likely attributed to benthic O_2_ uptake [6, 10, 38]—which brings up the difficulty to dissect the artificial flux from the true benthic flux. A response that cannot be imitated by the stirring sensitivity of the sensor is the gradual shift from negative fluxes (O_2_ consumption) to positive fluxes (O_2_ production) in environments where benthic photosynthesis changes the sign of the net flux [6]. However, even in this case the data has to be interpreted carefully, because the change of flow directions (e.g. tides) can also lead to a change from negative to positive artificial fluxes (compare Fig. 5 G+I). Thus care must be taken when light and flow directions co-vary.

**Figure 6:**
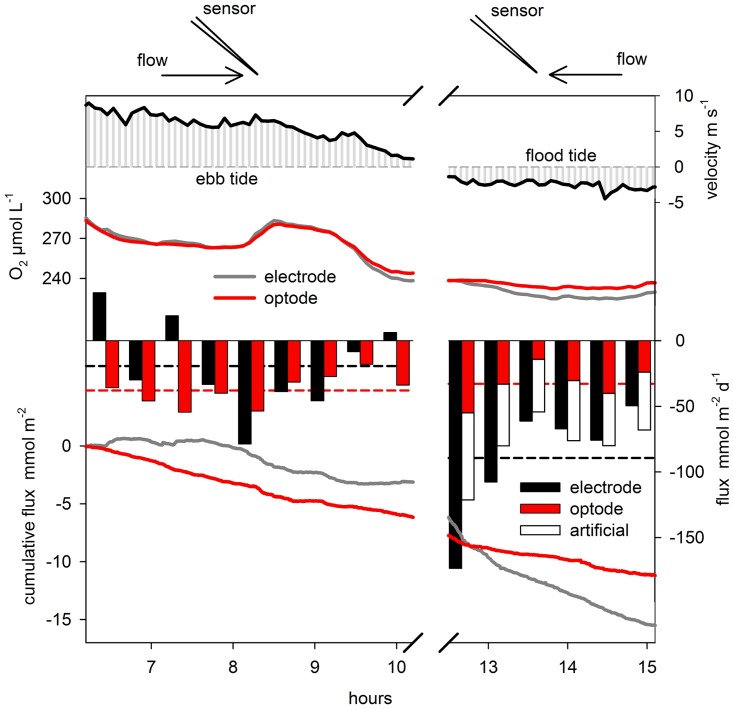
Dissecting true and artificial fluxes in field measurements. The figure shows an example of an EC-flux measurement in a tidally influenced fjord using optode and electrochemical sensors in parallel. Measurements during ebb tide and flood tide are compared (left versus right side), during which the sensor tip is first shaded from the flow and then facing the flow (see sketch above). Upper panels show the mean flow velocity and O_2_ concentrations. The bar plot shows the measured flux averaged over 25 minutes. The artificial flux was calculated using MODEL 2 and added to the optode flux. This was only possible during flood tide because the model could predict the vertical artificial flux only when the sensor is facing the flow (compare Fig. 5, sensor orientation ~60°). The dashed lines represent the average fluxes for the optode (red) and electrochemical sensor (black) during ebb tide and flood tide, respectively. The lower panel shows the cumulative flux. Processing of the continuously recorded data set was in the following order: despiking, planar fit rotation, mean subtraction with 60 s running average, time shift correction, and calculation of the mean flux for 25 minute intervals.

To evaluate field data, it is recommended to compare the predicted artificial flux from MODEL 1 or MODEL 2 with the EC-flux at a sensor orientation of ~40–60° (typical orientation). A prerequisite is of course the knowledge of the stirring sensitivity (*S_sen_*) of the O_2_ sensor, whereas the values for *B* (30 ± 4.9) and *n* (0.65 ± 0.03) can be adopted from this study. If the predicted artificial fluxes are in the order of the measured fluxes a significant bias from stirring sensitivity is likely. In principal it is possible to correct for the artificial flux at the typical sensor orientation. However, as the artificial flux in the field cannot be measured, but only be modeled any correction needs to be thoroughly vetted and additional indications for significant artificial fluxes would be helpful. Apart from the model predictions, artificial fluxes may be indicated when the measured fluxes change significantly with current direction. Fig. 6 shows a selected example of a field measurement using optode and electrochemical O_2_ sensors in parallel. The O_2_ flux was measured during the night in a tidally influenced shallow fjord (Odense Fjord, N 55°29’ E 10°29’). During ebb tide, the tip of the electrochemical sensor was shaded from the flow. During flood tide the sensor tip was facing the flow (compare Fig. 5A+C). Using the optode, the flux was fairly constant throughout the entire measurement showing similar mean fluxes at ebb and flood tide of -38 (± 12) and -33 (± 14) mmol m^-2^ d^-1^. However, using the electrochemical sensor, the flux at ebb tide was highly variable with occasional positive fluxes and a decreased mean flux of -19 (± 36) mmol m^-2^ d^-1^, whereas the mean flux increased to -89 (± 46) mmol m^-2^ d^-1^ during flood tide. Assuming that the flux from the optode measurement reflects the true benthic flux, the contribution of the artificial flux measured by the electrochemical sensor becomes apparent. The artificial flux at ebb tide was erratic but largely positive (comparable to Fig. 5I), which reduced the measurable true benthic flux and increased the variability. At flood tide, the artificial flux was negative (comparable to Fig. 5G) and added to the benthic flux, which resulted in the doubling of the measured fluxes compared to the benthic flux. At the latter sensor orientation, the artificial flux was predictable and could explain most of the differences found between electrochemical and optode sensors. It should be mentioned, that in subsequent deployments at the same site, optode and microelectrode derived data were better aligned and in other periods the discrepancy in derived fluxes from the two sensors could not be fully explained by stirring effects. The overall high benthic O_2_ flux and its high variability during changing light, current and bottom water O_2_ often complicated a clear distinction between true and artificial fluxes. This demonstrates the difficulty in assessing the contributions from the artificial flux in natural dynamic settings, but also that the impact may easily be overlooked especially when conditions and fluxes vary a lot.

It is difficult to know how much the stirring effect of microelectrodes has affected previously published EC fluxes. As outlined above, the importance can vary by orders of magnitude depending on the turbulence intensity, sensor orientation and sensor characteristics. Likewise, the relative contribution of the stirring induced signal can vary from negligible to dominant among studies. Some EC studies provide independent assessments of the benthic O_2_ exchange rates and reasonable alignment to average rates derived from chambers, O_2_ microprofiles, or mass balance calculations put a limit to how much the result could have been impacted. In recent studies, conducted in low turbulent environments, we and others have experienced good alignment between time-averaged EC fluxes as derived by parallel in situ deployments of microelectrode and microoptode (K. Hancke et al. unpubl. results) [19, 39, 40], indicating low contributions of artificial fluxes at low turbulent conditions. Such observations underpin the complexity of trying to quantify the contribution of the artificial flux to derived EC fluxes. For future studies we recommend the use of non-stirring sensitive optode sensors, especially in high turbulent environments, to exclude potential artifacts and avoid the challenging evaluation of artificial flux contribution.

## Conclusions

Theoretical constraints and experimental results clearly indicate that the stirring sensitivity of electrochemical O_2_ sensors can cause a significant correlation of flow velocity and apparent O_2_ signal, which is difficult to distinguish from the correlation found during ordinary turbulent O_2_ transport in bottom waters. For the typical sensor orientation, when the sensor is facing the flow, the resulting artificial flux is predictable and may range from less than 1 to more than 100 mmol m^-2^ d^-1^. The artificial flux is downward directed and increases with Reynolds stress (i.e. with current velocity and bed roughness), mean O_2_ concentration and the stirring sensitivity of the sensor. As a consequence, the artificial flux mimics the benthic O_2_ uptake and most of the expected responses to changing environmental conditions. For sensor orientations where the senor tip is shaded from the flow, the artificial flux is not predictable and behaves erratic. This dependence on the flow direction may be used for EC measurements as indicator for the appearance of artificial fluxes. However, we recommend discarding the flux measurement if the predicted artificial flux is in the range of the measured flux. Optical O_2_ microsensors (micro-optodes) were tested in parallel and showed no stirring sensitivity. For future studies, we recommend the use of optode sensors.
