# Correction: Aquatic Eddy Correlation: Quantifying the Artificial Flux Caused by Stirring-Sensitive O_2_ Sensors

**DOI:** 10.1371/journal.pone.0121931

**Published:** 2015-04-02

**Authors:** The PLOS ONE Staff

There are errors in equations (4) and (7) of the published PDF. Please view the correct equations here.

$$F=\frac{c_{\infty }}{\bar{u}}\frac{S_{sen}\;B\;{\bar{u}}^{n}n}{{\left(1+B\;{\bar{u}}^{n}+S_{sen}\right)}^{2}}$$ (4)

$$EC\;flux_{art}=\frac{c_{\infty }}{\bar{u}}\;\frac{S_{sen}\;B\;\;{\bar{u}}^{n}\;n}{{\left(1+\;B\;\;{\bar{u}}^{n}+S_{sen}\right)}^{2}}\;{\left(\frac{u\;\kappa }{\ln(z/z_{0})}\right)}^{2}$$ (7)
